# Emotions and Dog Bites: Could Predatory Attacks Be Triggered by Emotional States?

**DOI:** 10.3390/ani11102907

**Published:** 2021-10-08

**Authors:** Serenella d’Ingeo, Fabrizio Iarussi, Valentina De Monte, Marcello Siniscalchi, Michele Minunno, Angelo Quaranta

**Affiliations:** 1 Animal Physiology and Behavior Unit, Department of Veterinary Medicine, University of Bari Aldo Moro, 70100 Bari, Italy; marcello.siniscalchi@uniba.it (M.S.); m.minunno79@libero.it (M.M.); angelo.quaranta@uniba.it (A.Q.); 2 Veterinary Section, Department of Emergency and Organ Transplants, University of Bari Aldo Moro, 70100 Bari, Italy; fabrizio.iarussi@uniba.it; 3 Private Practitioner, 70121 Bari, Italy; valedemo@inwind.it

**Keywords:** dog bite, aggressive behavior, predatory behavior, emotions, physiology

## Abstract

**Simple Summary:**

Dog bites are a worldwide problem that have severe consequences for both the animal and the victim involved in the incident. Epidemiological studies have analyzed the victim features, the characteristics of biting dogs and the context in which attacks occur. Little is known regarding the role of emotions in predatory attacks toward humans and conspecifics in dogs. This paper aims at proposing the potential involvement of emotions for the expression of predatory motor patterns. It is suggested that the reporting of dog biting episodes needs to consider this crucial factor, which is fundamental for providing a realistic and reliable picture of the dog bite phenomenon.

**Abstract:**

Dog biting events pose severe public health and animal welfare concerns. They result in several consequences for both humans (including physical and psychological trauma) and the dog involved in the biting episode (abandonment, relocation to shelter and euthanasia). Although numerous epidemiological studies have analyzed the different factors influencing the occurrence of such events, to date the role of emotions in the expression of predatory attacks toward humans has been scarcely investigated. This paper focuses on the influence of emotional states on triggering predatory attacks in dogs, particularly in some breeds whose aggression causes severe consequences to human victims. We suggest that a comprehensive analysis of the dog bite phenomenon should consider the emotional state of biting dogs in order to collect reliable and realistic data about bite episodes.

## 1. Introduction

Dog bites are a serious worldwide problem. The consequences for human health include physical injuries, transmission of zoonosis and psychological trauma [1]. The issue of dog biting also has a significant impact on dog welfare. It is a common cause for abandonment, relocation to shelter and euthanasia [2,3]. Dog-related human fatalities are rare events (0.01% of all dog bites) but they are the most serious consequence of dog attacks [4]. More than 300 individuals died from dog attacks in the US from 1979 to 1998 [5]. In France, 20 bite-related fatalities have occurred in the past 30 years [6]. In Italy, from 1984 to 2020, 58 individuals died from dog attacks ([7]—data from media reports of 2009-2020). 

In recent years, many studies have been carried out in order to understand the epidemiology of dog bites. Several authors have investigated the victim features, the characteristics of biting dogs and the context in which attacks occur [3,8,9,10]. This led to the definition of a dog bite as a multifactorial phenomenon, whose expression is regulated by genetic, physiological, developmental, environmental and social factors [4]. According to the clinical and behavioral assessments of biting dogs, the attacks toward humans are most commonly caused by fear and anxiety (77%) [11,12,13,14], suggesting that dogs’ emotions and their relationship to humans are crucial components of the biting phenomenon. Recent studies have demonstrated, indeed, that dogs are able to interpret human emotional states and regulate their behavior accordingly [15,16,17,18]. Therefore, their influence on the development of dog biting events needs to be further and deeply investigated. 

This paper aims at analyzing the potential influence of emotions on dog aggressive behavior, with a specific focus on the importance of this factor for the expression of predatory aggression, particularly in some breeds.

## 2. Emotions and Aggressive Behavior in Dogs: The Role of Emotions in Biting Episodes

Dog aggressive behaviors are broadly divided in two main categories: predatory behavior and affective aggression [19]. They mainly differ in their aims and neural regulation. Predatory motor patterns are part of feeding behavior: they aim at obtaining food by killing and consuming prey [19,20,21]. In dogs, the predatory sequence includes different motor patterns (more generally defined as predatory behaviors): orienting towards prey, eye stalk, chase, grab bite, kill bite (or head shake), dissection and consumption [20]. In the literature, it is reported that dogs might engage in predation toward both conspecifics and heterospecifics (e.g., humans [19]). This behavior is widely described as “predatory aggression”. It has been considered a non-emotional or non-affective type of aggression, where communication between subjects is absent and the sympathetic arousal is low [19,22,23,24]. Conversely, social/affective aggression (which might be offensive and defensive) has a strong emotional and communicative component and is accompanied by a significant sympathetic activation [19,23,25]. Social aggression serves to increase the distance between subjects and eventually avoid/control negative outcomes through the expression of threatening behaviors (e.g., growling, posturing, snapping) [19,20,23]. Affective aggression is triggered by transient emotional states (e.g., frustration, fear, irritability, anxiety) [19]. Besides the differences in their scopes, the neural regulation of predatory and affective aggression also involves distinct structures of the hypothalamus: the ventro-medial hypothalamus controls affective aggression, whereas the lateral hypothalamus regulates predatory behavior [19]. Both of them receive inputs from the limbic system, specifically from the amygdala [23,26]. 

Recently, some authors challenged the inclusion of predatory attack into the general category of aggressive behavior, which includes, for instance, play-related aggression, territorial aggression, defensive aggression and fear-related aggression [23,26,27,28,29]. Indeed, aggression is defined as “threats, postures, or harmful actions directed towards another individual. Aggression is a form of communication, where aggressor is attempting to establish greater social distance between himself and the target of his aggression” ([20], p. 2). It is also “one component of agonistic behavior that serves to regulate individuals’ ability to compete for various resources (food, shelter, territory, mates, social status)” [4]. Moreover, it has been defined as “an appropriate or inappropriate threat or challenge that is finally resolved in an act of fight or disengagement [29,30]” as well as “a behavior or model of threatening and confrontational behaviors used to resolve a conflict, which is finally settled through confrontation or backing/withdrawal [30,31]”. In predatory “aggression”, which includes behaviors aimed at capturing and killing prey, the intent to communicate and challenge other individuals as previously described is lacking. We therefore believe that it would be more appropriate to refer to predatory behavior causing lesions to other individuals as “predatory attack” rather than predatory aggression. We will refer to this phenomenon by using these terms.

While the involvement of emotions in the expression of social aggression is clearly described and broadly recognized, their influence on predatory attacks was at first excluded (as reported above). However, available and recent evidence suggests that emotional states might have a role in triggering predatory attacks in dogs. Some authors showed that predatory attacks, which usually cause severe injuries or the death of the victims, could be influenced by emotional factors, such as anxiety or frustration, which modulate their expression [19,24]. Among the main causes of dog killing aggression toward humans and conspecifics is an insufficient socialization with these species, and previous unpleasant or traumatic experiences (the “aggressors” were previously victims of a conspecific aggression or attack) could play an important role [24,25]. They might lead dogs to experience emotions such as fear, anxiety and frustration during social encounters, which increase the subjects’ emotional tension (stress) and might consequently trigger the expression of predatory behaviors [25]. Frustration, indeed, is generally triggered by unpredictable and uncontrollable events that elicit contrasting emotions and inner conflicts [19]. It has also been previously related to the engagement of the reactive aggression (RAGE) system [32], which has been anecdotally described for backyard dogs due to the presence of barriers or any forms of confinement [33]. It causes the increase in subjects’ arousal, which is functional for identifying an adaptive response when facing an unexpected event [19]. However, when the arousal and frustration levels are too high, the emotional tension might persist and lead to the expression of behaviors in an intense and dysregulated way. As a consequence, the subject could engage in behaviors aiming at restoring the emotional homeostasis. 

An important role for the regulation of stress-related behavior is played by dopamine. It has been found that its concentration decreases in the dopaminergic mesolimbic system (“reward system”) in response to stress, arousal increase and in the presence of environmental alarming signals [34]. Dopamine has a key role for the coordination and regulation of motivated behaviors: the reward system is activated when motivations are fulfilled. This leads dogs to experience pleasant emotions and the achievement of pleasure [35]. We can therefore hypothesize that, when experiencing an emotional conflict (i.e., frustration) or an increase in their emotional tension, subjects could engage in highly gratifying and motivated behaviors for restoring their emotional homeostasis by the activation of the reward system. The behaviors expressed might not be adaptive per se (i.e., removing the stressor that generated the conflict) but they might only have the aim of decreasing the emotional tension in individuals.

Among the highly motivated behaviors, predatory motor patters are particularly rewarding for dogs. Their expression, indeed, maximally activates the reward neural centers [24] and generates a pleasant hedonic experience, which is analogous to the satisfaction of hunger and thirst [19]. It could be possible, therefore, that dogs could engage in predatory behaviors when experiencing stress, frustration and an arousal increase in order to reach pleasure and gratification, which could subsequently reduce the state of emotional tension generated by a stressor. 

Evidence for an association between the arousal increase and the expression of predatory attacks has also been shown in a recent study. Schilder and colleagues [25] found that dogs, which previously showed predatory attacks toward other conspecifics, expressed high vigilance during the walk with the owner even before meeting with another dog. The subjects’ arousal significantly increased at the sight of a conspecific or when they reached a place where they expected to meet another dog (i.e., anticipation). 

In light of this, we could hypothesize that predatory attacks could be triggered both by the movement of the victim (i.e., prey) but also by a state of emotional tension, which might be “discharged” through the expression of highly rewarding predatory behaviors. This could make the treatment of this specific behavioral problem very difficult, as previously reported [24].

However, we cannot rule out the possibility that the arousal increase registered before the expression of predatory attack could be related to the anticipation of positive outcomes, i.e., food consumption. Therefore, further studies are needed to clarify the cause of subjects’ arousal increase in order to understand its valence (i.e., positive or negative) and to measure its intensity, by evaluating dog behavior and other physiological parameters (e.g., heart rate increase by ECG wireless systems; body and facial surface temperature [16,17,18,36]). Similarly, since frustration has been implicated in the expression of displacement and repetitive behaviors [37,38,39], it could be possible that one or more phases of the predatory attack triggered by frustration could be an expression of displacement activities, which are usually normal behaviors expressed in an inappropriate context and time [20].

It has been shown that predatory attacks could be more easily triggered in subjects showing predatory motor patterns towards all type of animal species [24,25]. The genetic selection of different breeds has a deep influence on dog phenotypical and behavioral features. Humans modified and differentiated the motivations of different breeds according to their role in human society [23]. For some breeds, humans selected and emphasized specific phases of the predatory sequence for their work, increasing the likelihood of their expression and their rewarding value. Headers (including border collies), for instance, were selected for conducting herds by causing them no injuries. The behavior of stalking and chasing was hypertrophied while the bite (both grab and kill bite) was diminished. Similarly, livestock guardian dogs (such as the Maremma sheepdogs) were selected for the absence of any predatory behavior directed toward the sheep; therefore, the entire predatory sequence was “quieted” [20,40,41]. Different breeds were also selected for dog fighting, which was highly popular in the 1800s [42]. They include “pit bull” dog types, bull terriers, mastiffs and bull dogs [43] (later referred to as “fighting dog breeds”), which show similar phenotypical and behavioral characteristics. In particular, the bite behaviors belonging to the predatory motor patterns (i.e., sustained grabbing, holding, shaking, tearing) were selected and emphasized in order to produce the maximum damage to the dog opponent during fighting [44]. Since genetic selection has also acted on the functionality of the nervous system by affecting the distribution of dopamine receptors [23], it is likely that the expression of the selected hypertrophied behaviors would significantly increase the dopamine levels in the reward centers and consequently intensely gratify dogs. Therefore, it could be possible that dogs belonging to the breeds where specific phases of the predatory sequence were hypertrophied may engage in predatory behaviors during stressful situations in order to restore their emotional homeostasis and experience pleasure. In other words, when a border collie is experiencing a high arousal increase (related to stress or frustration) it could be likely that it engages in chasing objects or other individuals, whereas “fighting dog breeds” might direct predatory bites towards objects, conspecifics and even humans. This might pose a severe risk for humans and public health. Indeed, pit bulls (even though they do not belong to an officially recognized breed) are frequently involved in biting episodes worldwide and particularly in the US and UK [45,46]. “Fighting dog breed” (including pit bulls) selection also fixed behavioral and personality characteristics that were useful their work [4,44]:

− Gameness: high perseverance until the goal is reached, causing the lack of sensibility toward the other subject’s surrender signals;
− Low inhibition for fighting: high reactivity to minimum threats (moving or non-moving stimuli) activates behavioral responses until the complete exhaustion or death;
− Low sensitivity to pain;
− Scarce communication, which enhances the unpredictability of the attack.

The lack of prodromal warning signals (e.g., growl, freeze, snap, directed and prolonged gaze) preceding the attack, indeed, is frequently reported by human victims [4,47,48,49]. Breeding has also caused a genetic vulnerability due to a greater sensitivity to threatening stimuli [25], which might be related to a different perception of environmental and social stimuli [23].

It is possible therefore that an environmental/social stressor may cause a significant increase in these breeds’ emotional tension (due to their high sensitivity) that could be “discharged” by the expression of the predatory bite, which produces a deep sense of gratification and pleasure in these dogs.

The sensitivity and vulnerability to stress, along with the high perseverance and the lack of responsiveness to the opponent’s reactions, as well as the high gratification produced by the expression of the kill bite (aiming at inflicting the maximum damage to the victim), make the attack of “fighting dog breeds” particularly dangerous, and raise serious issues for public health.

Although this might explain the high prevalence of severe/fatal attacks of pit bulls reported by the recent literature and media, some important considerations about this phenomenon are needed. Firstly, numerous mixed-breed dogs are referred to as pit bulls on the basis of their phenotypical similarities. It is widely reported, indeed, that the ability of the public, the victims and official authorities to correctly identify dog breeds and dangerous dogs is notoriously faulty [50,51]. This could lead to a significant overestimation of the involvement of pit bulls in biting events. It could also be affected by the general lack of demographic data regarding the breed representation within the general registered dog population [4,44]. Therefore, despite the removal of “fighting” dogs from the breeding of officially recognized breeds, which aims at mitigating the personality characteristics of these dogs, the lack of reliable data about the involvement of these dogs in biting episodes makes the evaluation of the influence of genetic factors particularly difficult [44]. “Fighting dog breeds”, indeed, have also been selected for stability and tractability with people [52] and make the dogs belonging to officially recognized breeds suitable as household companions [42]. However, detailed data are instead necessary in order to assess and monitor the long-term effect of the current rebreeding process of officially recognized “fighting dog breeds” on their personality traits. 

In light of this, and considering the multifactorial nature of dog bites, it is crucial that the behavioral assessment of biting dogs is carried out by behavior specialists in order to evaluate the different motivations and emotions that trigger aggression, as well as the influence of individual personality and the social and environmental living conditions on the expression of such phenomena. This specific and comprehensive evaluation is necessary for a correct management and application of a therapeutic plan that needs to be individual oriented.

## 3. Conclusions

Emotions play a pivotal role in the development and expression of dog behavior, including aggression and predatory attacks towards humans or conspecifics. Therefore, a comprehensive analysis of all the factors involved in the onset of biting events should not disregard the evaluation of dog emotional state, as well as the relationship with the owner and the member of the familiar group, as well as the management and the living conditions of the animal. This would allow veterinarians and researchers to collect a realistic and reliable picture of the dog bite phenomenon. 

## Data Availability

Not applicable.
